# The macromolecular crystallography beamlines of the Helmholtz-Zentrum Berlin at the BESSY II storage ring: history, current status and future directions

**DOI:** 10.1107/S1600577525001110

**Published:** 2025-03-31

**Authors:** Uwe Mueller, Tatjana Barthel, Laila S. Benz, Volodymyr Bon, Thomas Crosskey, Camilla Genter Dieguez, Ronald Förster, Christine Gless, Thomas Hauß, Udo Heinemann, Michael Hellmig, David James, Frank Lennartz, Melanie Oelker, Ruslan Ovsyannikov, Parinita Singh, Markus C. Wahl, Gert Weber, Manfred S. Weiss

**Affiliations:** a Macromolecular Crystallography Helmholtz-Zentrum Berlin für Materialien und Energie Albert-Einstein-Str. 15 12489Berlin Germany; b Fachbereich Biologie, Chemie, Pharmazie, Institut für Chemie und Biochemie Freie Universität Berlin Takustr. 6 D-14195Berlin Germany; c Fakultät Chemie und Lebensmittelchemie Technische Universität Dresden Bergstr. 66 01069Dresden Germany; d Max-Delbrück Centrum für Molekulare Medizin Robert-Rössle-Str. 10 D-13125Berlin Germany; e Humboldt-Universität zu Berlin Unter den Linden 6 D-10099Berlin Germany; f IT-Systems and Technology Helmholtz-Zentrum Berlin für Materialien und Energie Albert-Einstein-Str. 15 D-12489Berlin Germany; Cornell University, USA

**Keywords:** macromolecular crystallography beamlines, synchrotron radiation, automation, high-throughput crystallography, crystal dehydration, crystallographic fragment screening, large-scale facility, chemical crystallography, user operation, cyber attack

## Abstract

Since 2003, the Macromolecular Crystallography (MX) group at Helmholtz-Zentrum Berlin (HZB) has been operating three MX beamlines at the BESSY II storage ring in Berlin. These beamlines were established to support the emerging structural genomics initiatives founded in Germany, Europe, and overseas around the turn of the century. Over the past two decades, these beamlines have been continuously developed to enable state-of-the-art diffraction experiments and to provide supporting facilities such as a sample preparation laboratory, a spectroscopy laboratory, a Biosafety Level 1 laboratory and all necessary computing resources for the MX and chemical crystallography user community. Currently, more than 100 independent research groups from the greater Berlin area, Germany, and Europe utilize these beamlines. Over time, more than 4500 Protein Data Bank depositions have been accrued based on data collected at the beamlines. This paper presents historical aspects of the beamlines, their current status including their research output, and future directions.

## Abstract 2

The history, current state and future directions of the three MX beamlines BL14.1, BL14.2 and BL14.3 of the Helmholtz-Zentrum Berlin at the BESSY II electron storage ring are described.

## 1. Introduction

### 1.1. Current state of macromolecular crystallography

**Figure 1:**
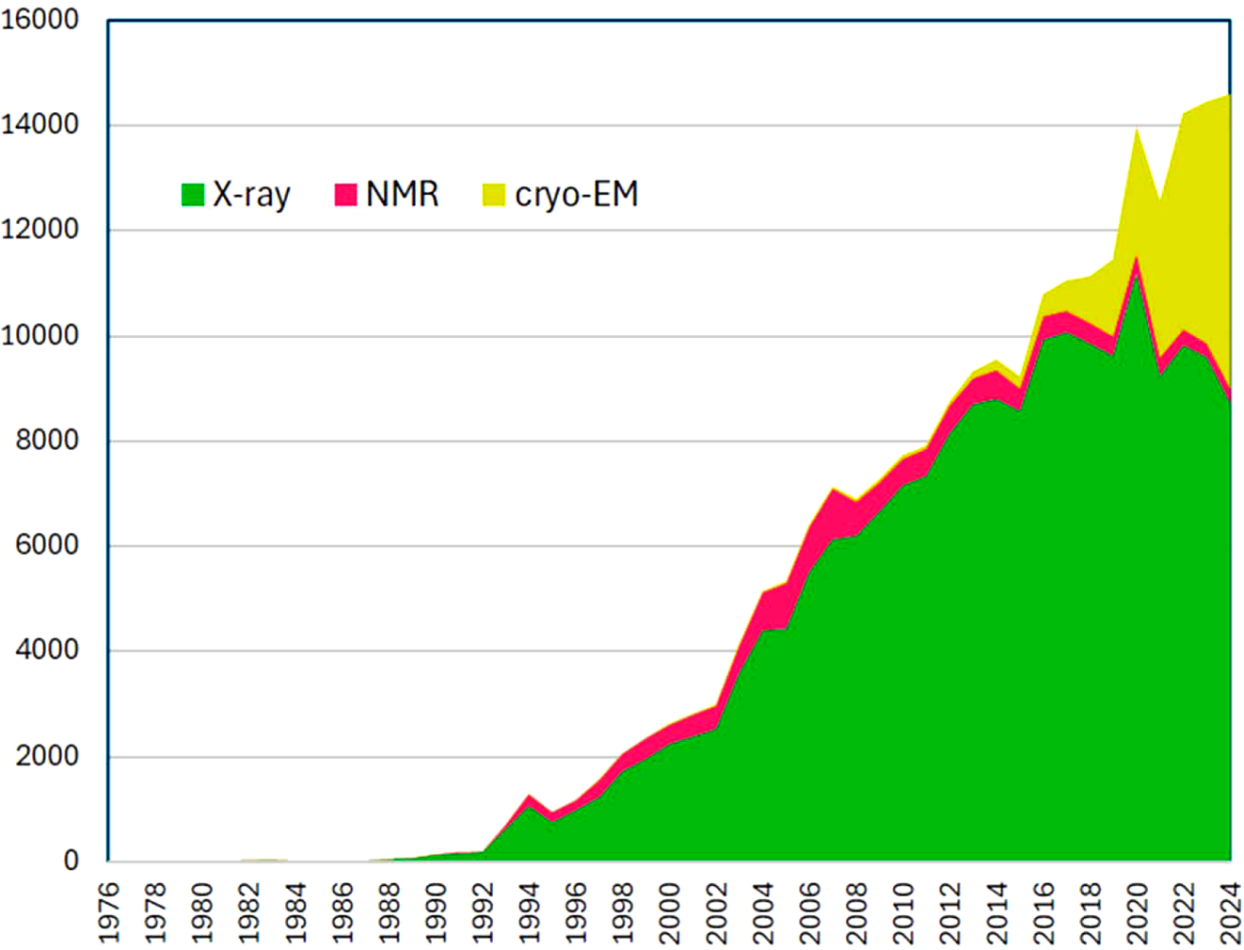
PDB growth by method as of 10 December 2024. (Note: the numbers for 2023 and 2024 will only be final at the end of 2024 and 2025, respectively, due to the one-year hold period policy of the PDB.)

The field of macromolecular crystallography (MX) has undergone dramatic developments in the last decade. In the mid-2010s, advances in cryo-electron microscopy (cryo-EM) brought about the resolution revolution (Kühlbrandt, 2014 ▸), which established cryo-EM as an alternative method for structure determination at a resolution sufficient to fit atomic models. Structures of large multi-protein complexes and of membrane proteins, which are both notoriously hard to crystallize, could now be tackled by cryo-EM. In the early 2020s, there was the transformative release of *AlphaFold2* (Jumper *et al.*, 2021 ▸), which enabled the prediction of a 3D structural model for almost every possible sequence of amino acid residues. Despite all these developments, MX was and still is the dominant method, in terms of the number of deposited structures per year, for the structure determination of proteins, protein/nucleic acid complexes and protein/ligand complexes. Moreover, over the past 50 years, MX has contributed more to our understanding of the molecular principles of life than any other structure determination technique as shown by the current content and the growth statistics (Fig. 1 ▸) of the Protein Data Bank (PDB) (Berman *et al.*, 2000 ▸).

As of 10 December 2024, more than 228000 structures of the molecules of life have been determined experimentally and deposited in the PDB, of which about 190000 (83%) have been determined by MX. This enormous wealth of information is used by an ever-growing community of researchers to address important questions in biochemical, molecular biological and biomedical research. It is also clear that the success of *AlphaFold2* and related structure prediction methods is based on the availability of these experimental protein structures assembled and well curated in the PDB. Despite the enormous growth of cryo-EM and the advent of *Alphafold2*, the number of MX structures deposited per year in the PDB is not decreasing. Clearly, due to *Alphafold2*, experimental phase determination experiments have become largely obsolete, but it is also clear that a predicted model alone without any experimental structure information is not (yet) sufficiently accurate to help gain a deeper understanding of the structure–function relationship of the protein or protein complex under investigation (Terwilliger *et al.*, 2024 ▸). As a consequence of the reduced demand for experimental phase determination experiments, the MX community can focus on other experiments, such as crystallographic fragment screening (CFS) (Barthel *et al.*, 2024 ▸; Fearon *et al.*, 2025 ▸; Kanchugal *et al.*, 2025 ▸) or other multi-crystal experiments such as radiation dose series, concentration series *etc*. Also, the discovery of significant conformational differences between structures determined at 100 K and structures determined at ambient temperature led to the re-emergence of room-temperature crystallography (Fraser *et al.*, 2011 ▸). Consequently, we anticipate that there is no less demand for synchrotron beamtime than before cryo-EM and *Alphafold*, and the almost 100 MX beamlines, which are available to the user community worldwide, will continue to be busy.

### 1.2. History of the HZB-MX beamlines

**Figure 2:**
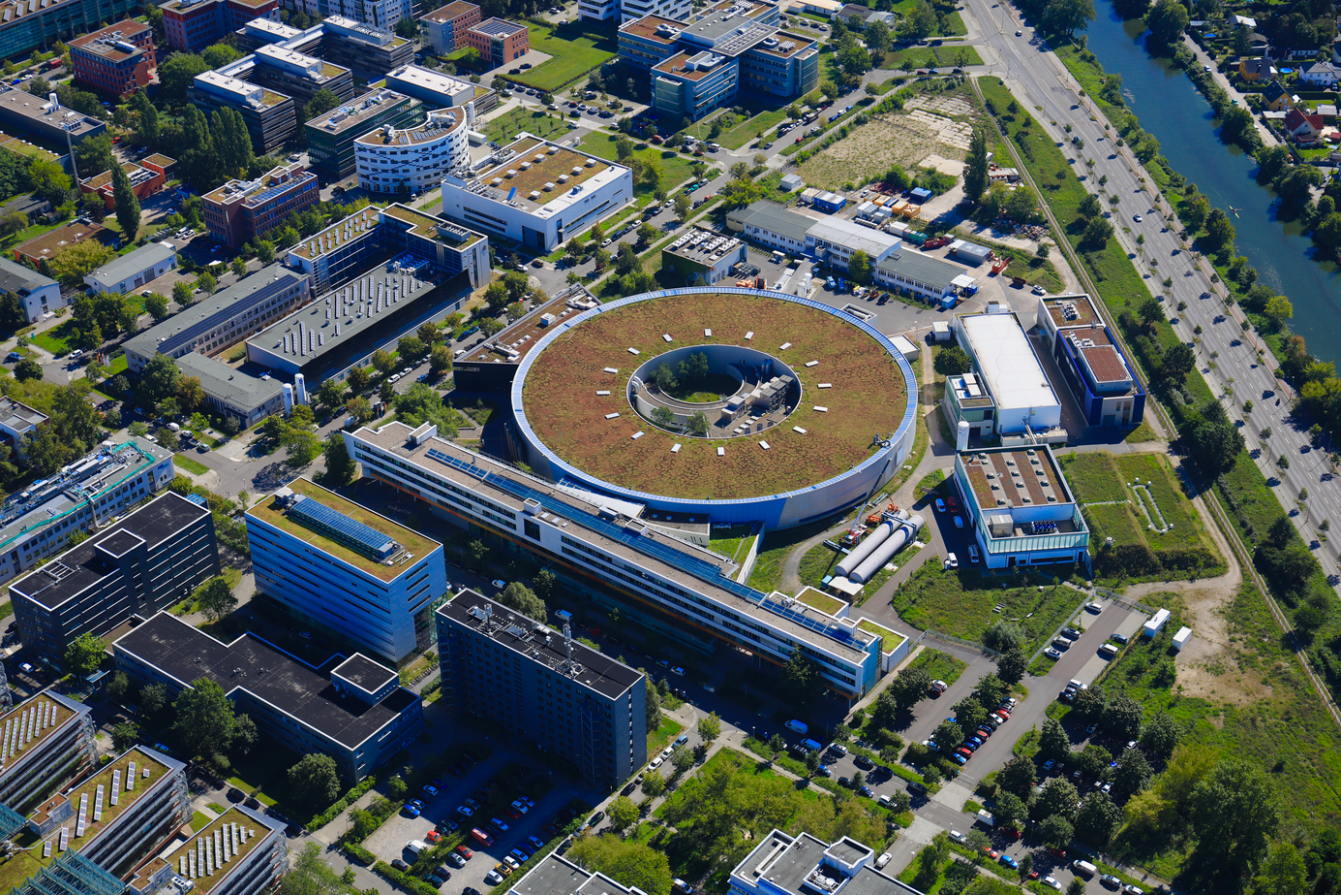
Aerial view of the BESSY II electron storage ring in Berlin-Adlershof, Germany.

The MX beamlines at BESSY II were initially conceived to serve the projected needs of a Berlin-based structural genomics consortium named the Protein Structure Factory (PSF) (Heinemann *et al.*, 2000 ▸). Around the turn of the millennium, structural genomics projects aiming at high-throughput, large-scale protein structure determination were initiated in Asia (Kigawa *et al.*, 2000 ▸), North America (Terwilliger, 2000 ▸) and Europe (Heinemann, 2000 ▸). They were stimulated by predictions that the total number of protein folds is limited and orders of magnitude smaller than the number of protein-coding genes in the biosphere and that it should thus be feasible to generate useful structural models covering a large fraction of protein fold space using a systematic target selection and structure analysis approach (Vitkup *et al.*, 2001 ▸). Deciphering the 3D structures for most of these protein folds was expected to greatly facilitate and accelerate further experimental structure determination, computational structure prediction and, ultimately, structure-based drug development. However, this gargantuan task required a concerted international effort towards improving technologies, creating facilities and achieving standardization of research tools. With this goal in mind and anticipating future use by a wider structural biology community beyond structural genomics, the Berlin PSF project set out to generate methods and instrumentation for high-throughput protein production, protein characterization, crystallization and structure analysis by NMR and X-ray diffraction (Heinemann *et al.*, 2003 ▸). The latter included the current MX beamlines, which were established at the then recently opened BESSY II synchrotron in Berlin-Adlershof (Fig. 2 ▸). The MX beamline sub-project of the PSF was planned and directed at the Max-Delbrück Center for Molecular Medicine, Berlin, and later transferred to Freie Universität Berlin, largely for administrative reasons. Since it was expected that most of the anticipated protein structure analyses would require experimental phasing through anomalous diffraction, the initial design put an emphasis on tuneable beamlines.

## 2. The HZB-MX beamlines

### 2.1. The BESSY II storage ring

BESSY II is a third-generation synchrotron facility operated by the Helmholtz-Zentrum Berlin für Materialien und Energie. It is located in the southeast of Berlin close to the Berlin-Brandenburg airport BER. The BESSY II machine has been in operation since 1998 and works at a ring energy of 1.7 GeV, thus enabling a broad experimental portfolio that spans from IR to VUV and XUV, and further into the hard X-ray energy range. It currently supports more than 50 beamlines. Among these are the three MX beamlines located in section 14 of BESSY II and named BL14.1, BL14.2 and BL14.3. During regular multi-bunch operation, the ring current is 300 mA, with regular topping up to compensate for electron beam loss.

### 2.2. The radiation source

The radiation source for the three MX beamlines is a superconducting 7 T wavelength shifter (7T-WLS), developed and constructed at the Budker Institute of Nuclear Physics in Novosibirsk, Russia, which was installed in the low-beta section of sector 14 of the BESSY II ring lattice in 2001. It has a broad horizontal radiation fan of 80 mrad and a very narrow vertical divergence of 0.1 mrad and a moderate source size of 50 µm×20 µm (h×v FWHM). This insertion device was designed to serve as the radiation source for all three HZB-MX beamlines, providing a critical energy of 13.5 keV at full field operation. During the more than two decades of operation, this device proved to be exceptionally reliable, operating for over 24 years without any unexpected downtime. In 2018, the 7T-WLS received an on-site major overhaul by the manufacturer. The upgrade included a new vacuum chamber, a new and highly efficient cryogenic design to minimize heat dissipation effects, new superinsulation seals and new control system hardware. Currently, the BESSY II machine group is investigating the possibility of replacing this insertion device by a functionally similar installation from another supplier to mitigate hardware issues that could arise in the future.

### 2.3. The three MX beamlines

The three HZB-MX beamlines (Mueller *et al.*, 2012 ▸; Mueller *et al.*, 2015 ▸) have now been in operation for over two decades. Their availability has significantly influenced the MX community in Berlin, Germany, and across Europe, as evidenced by more than 1500 publications and more than 4500 PDB depositions using data gathered at the beamlines. Despite being a slightly dated infrastructure when compared with more recent installations in Europe, it remains among the most productive MX facilities in Europe (see Biosync statistics https://biosync.rcsb.org). The reliability and productivity of the beamlines are due to continuous upgrades to the endstations, including sample changer robotics, modern detectors and goniometry. In addition, a loyal and growing user community has been built, which remains very active in utilizing the HZB-MX beamlines for their research.

**Figure 3:**
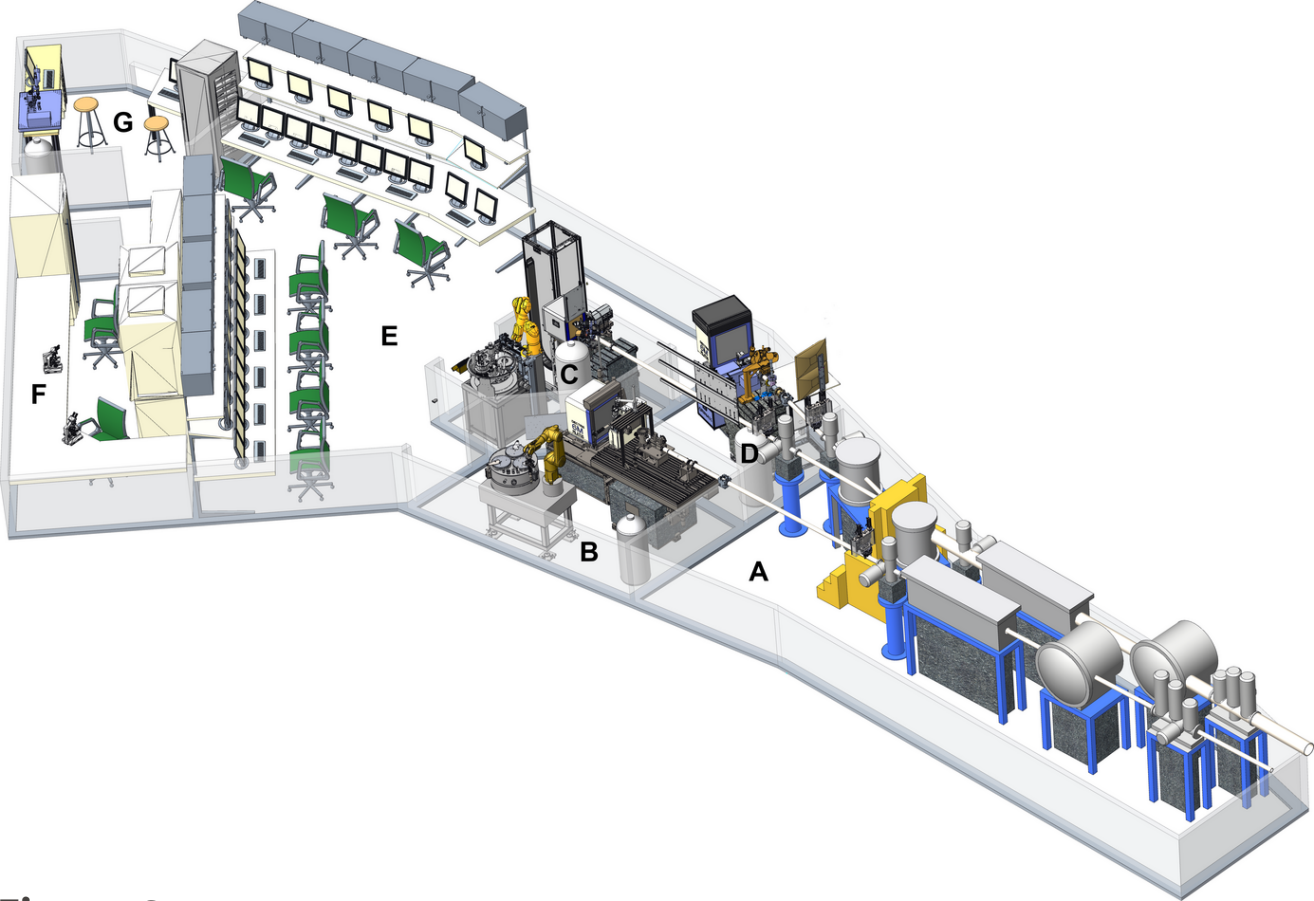
Schematics of the three HZB-MX beamlines. A: Shared optics hutch; B: BL14.1 experimental hutch; C: BL14.2 experimental hutch; D: BL14.3 experimental hutch; E: user operation center; F: sample preparation laboratory; G: SpectroLab.

The three beamlines feature a joint optics hutch and one experimental hutch per beamline. This enables the beamlines to be operated separately and independently from one another. The beamlines are operated by on-site users from a joint operations hutch (Fig. 3 ▸). Close to the operations hutch, users can access a sample preparation laboratory as well as a UV/Vis spectroscopy laboratory (MX-SpectroLab).

#### 2.3.1. BL14.1

BL14.1 is a 26 m-long beamline, which receives a 2 mrad cone, centered at −40 mrad inward of the ±40 mrad radiation fan of the 7T-WLS. It includes the following optical elements from upstream to downstream: a Rh-coated, 1200 mm-long, upward-deflecting, vertically collimating cylindrical silicon mirror; a water-cooled Si(111) double-crystal monochromator with a sagittally bent second crystal for horizontal focusing (AXILON, Germany); and a Rh-coated, 1200 mm-long, downward-deflecting, vertically focusing cylindrical glass mirror. This configuration produces a beam cross section at the sample position of 250 µm×100 µm (h×v FWHM). With the commonly used MD2 overfilling aperture of 50 µm diameter, the beam cross section is reduced to 60 µm × 40 µm (Mueller *et al.*, 2015 ▸). The beamline can be operated in the energy range 5.5–16 keV.

**Figure 4:**
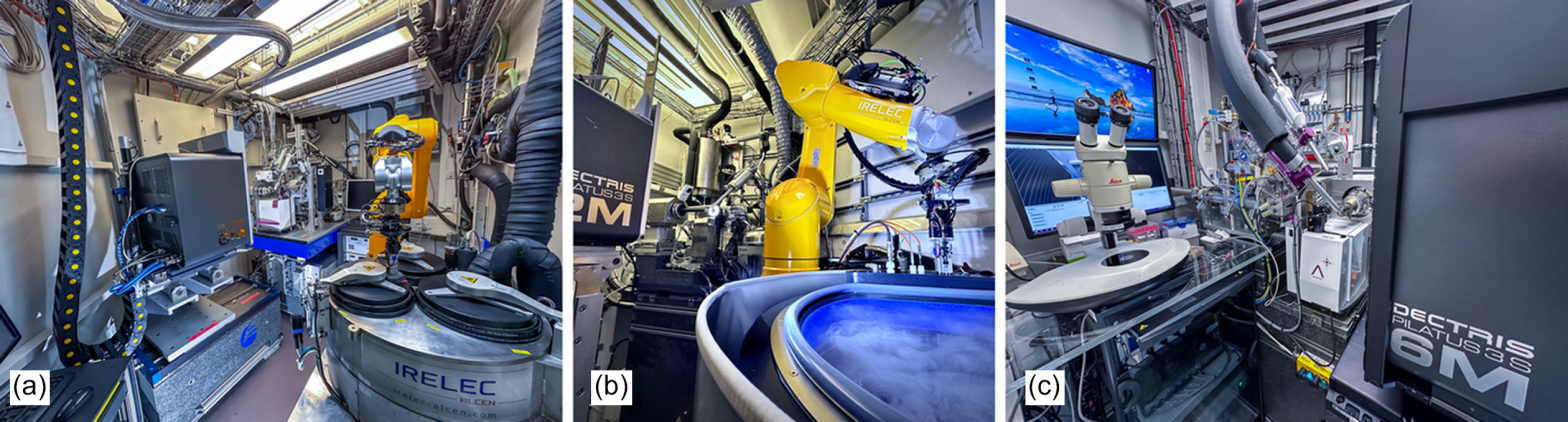
View of the endstations of BL14.1 (*a*), BL14.2 (*b*) and BL14.3 (*c*).

The experimental endstation of BL14.1 [Fig. 4 ▸(*a*)] includes an MD2 microdiffractometer with a minikappa goniometer MK3 (ARINAX, France), offering a small sphere-of-confusion (SOC) of approximately 1 µm RMSD. A Pilatus3X6M hybrid photon counting area detector (DECTRIS, Switzerland) offers a 100 Hz frame rate and provides a large 2Θ-acceptance range of 60° at a minimal achievable detector distance of 129 mm. Standard exposure times currently range from 0.03 to 0.3 s per frame. The setup is completed by a CATS sample changer with a 144-sample LN2 dewar (IRELEC-ALCEN, France), which is fully SPINE compatible and uses universal pucks (UniPucks) and a double gripper for dry sample transfer. On average, sample exchange times are in the range of 35 s. The sample changer has been in operation since 2008 and is to date one of the oldest systems of its kind still in operation. This setup allows for high throughput (HT) screening of up to 100 crystals within an 8 h user shift. The reliability of the sample transfer has been continuously improved and is now below 0.05% sample loss. Actual sample losses occur mainly due to non-standard or defective sample holders inserted into the system by beamline users.

At BL14.1, an AMPTEK XR-123SDD silicon drift diode detector is used for absorption-edge energy scans, typically requiring about 5 min per scan. Energy-dispersive fluorescence spectra analysis is also possible to identify the unknown metal content of proteins. BL14.1, along with all other beamlines, is controlled by VME hardware using *SPEC* (https://www.certif.com) and *TANGO* (https://www.tango-controls.org).

#### 2.3.2. BL14.2

BL14.2 is a 28 m beamline that receives the central 2 mrad cone of the ±40 mrad radiation fan of the 7T-WLS. Similar to BL14.1, the optical components include two Rh-coated, 1200 mm-long, cylindrical mirrors: one upstream of the monochromator system for vertical collimation and one downstream of the monochromator for vertical focusing. It utilizes the same tuneable Si(111) double-crystal monochromator system (AXILON, Germany) as BL14.1 and is able to achieve the same energy range (Table 1 ▸). Using beam finders and digital cameras installed in three different positions in the beamline, it is possible to monitor the dimensions and position of the beam during optimization. It partially shares a vacuum system with BL14.3, which diverges after the monochromator chamber. The beam cross section at the sample position is 150 µm×100 µm (h×v FWHM). The beam diameter is defined by the final 100 µm aperture with a resulting beam cross section of 110 µm × 60 µm.

The experimental endstation of the beamline [Fig. 4 ▸(*b*)] is equipped with an on-axis sample microscope camera (ARINAX, France) with user-controlled 12× optical magnification and a single-axis diffractometer with a SOC smaller than 100 nm, which was constructed by the HZB-MX group based on drawings provided by Alke Meents (DESY, Hamburg), while the beam shaping devices are attached to SLC linear piezo motors (SmarAct, Germany). The X-ray detector is a hybrid photon counting Pilatus3S2M (DECTRIS, Switzerland) with a 25 Hz maximum frame rate. The detector can be positioned only 57 mm from the sample, which combined with a higher X-ray energy at 15.5 keV allows high-resolution data collection up to 0.71 Å, also making the beamline suitable for small-molecule crystallography.

**Figure 5:**
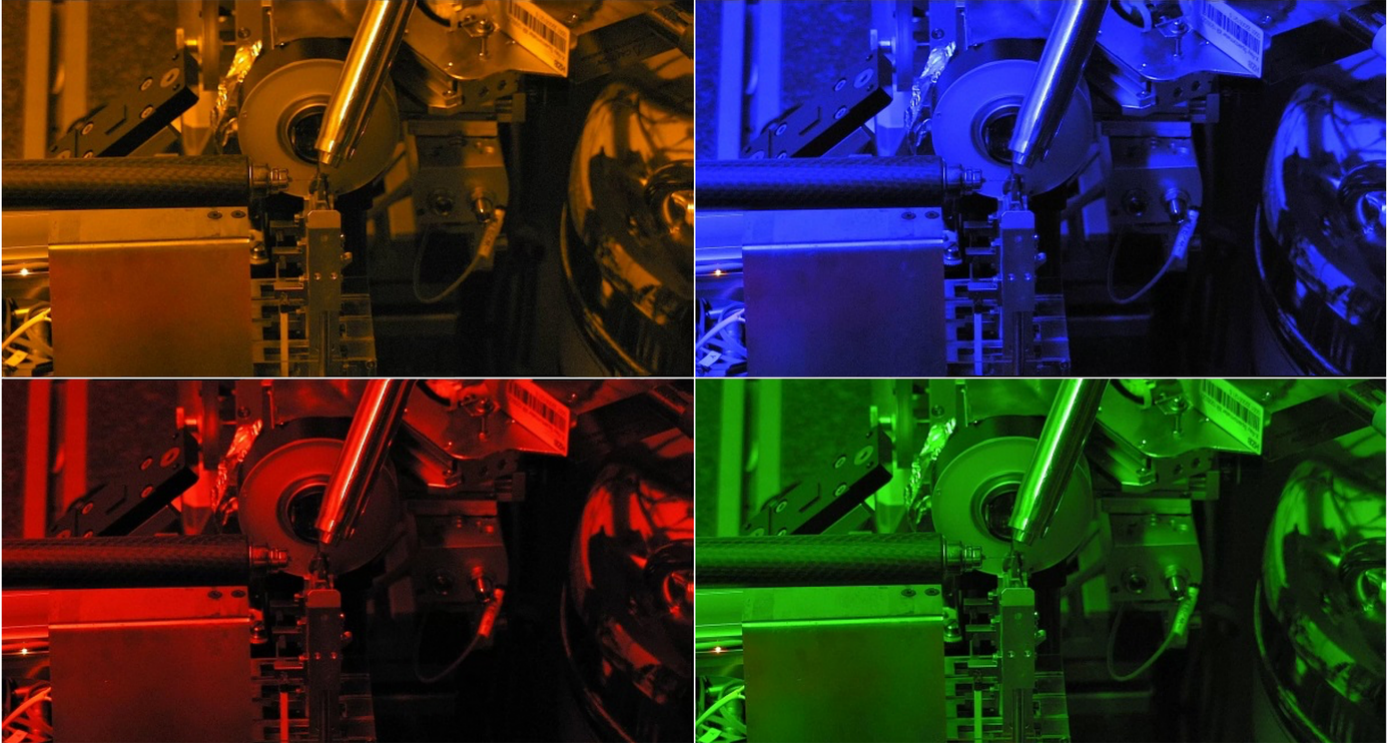
Safe light operation at BL14.2.

Like BL14.1, BL14.2 has an AMPTEK XR-123SDD Si drift diode detector mounted, which allows X-ray fluorescence experiments to be run. The BL14.2 experimental hutch is also equipped with narrow-bandpass LED adjustable ambient lighting, which allows the measurement of photo-sensitive crystals. Four conditions are available: red (629 nm), amber (591 nm), green (517 nm) and blue (462 nm) (Fig. 5 ▸). The ISARA2 robotic sample changer (IRELEC-ALCEN, France) was recently installed in the experimental hutch, which allows the automated mounting of up to 464 samples from 29 UniPucks. The average time to unmount a sample and mount the next is 15 s.

#### 2.3.3. BL14.3

Beamline BL14.3, extending 25 m from the X-ray source, operates with a static focus and maintains a constant energy of 13.8 keV. It partially shares its vacuum system with BL14.2 and is horizontally offset by 5 mrad in a counterclockwise direction relative to BL14.2. The optical components include an asymmetrically cut single-crystal Si(111) monochromator with direct water cooling, which can be meridionally bent for horizontal focusing (AXILON, Germany). Vertical focusing is achieved using a horizontally deflecting, lateral gradient, cylindrically shaped Si/Mo multi-layer mirror. Both optical elements are configured in a Kirkpatrick–Baez geometry. This configuration produces a beam cross section at the sample position of 300 µm×100 µm (h×v FWHM). Using a 100 µm-diameter aperture, the resulting beam cross section at the sample is 100 µm × 80 µm.

The endstation of BL14.3 [Fig. 4 ▸(*c*)] consists of an MD2-goniometer (ARINAX, France) with four different apertures of 50, 70, 100 and 200 µm and an Aerotech (AEROTECH, USA) stage that carries a Pilatus3S6M detector (DECTRIS, Switzerland). In standard operation mode, crystals are cryo-cooled at 100 K (Oxford Cryosystems, United Kingdom). A nozzle exchanger installed at the beamline rapidly switches between a 100 K LN2 cryo-stream and a HClab crystal humidifier (ARINAX, France) providing a lot of flexibility to the users. The crystal humidifier provides a controlled-humidity environment to a crystal, replicating the relative humidity in the original crystallization droplet (Kiefersauer *et al.*, 2000 ▸; Bowler *et al.*, 2015 ▸). Primarily, the device is very useful to assess crystals’ native diffraction capacity, irrespective of any effects of cryo-protectant that may be detrimental to diffraction. It can also be used for establishing humidity gradients and one further advantage is the option to conduct experiments at 100 K without any cryo-protectant after the complete removal of the surrounding mother liquor under the humidity stream at room temperature (RT) (Malinauskaite *et al.*, 2014 ▸; Monecke *et al.*, 2015 ▸; Klingl *et al.*, 2015 ▸). A summary of all relevant beamline parameters is given in Table 1 ▸.

### 2.4. Beamline development projects

Owing to their rather modest photon flux, the HZB-MX beamlines are at a disadvantage compared with other facilities regarding sample throughput. To partially mitigate this limitation, BL14.1 will soon be profoundly upgraded to a fixed-energy, large (1%) energy bandpass operation scheme (pink beam operation). This modification will increase the photon flux substantially, which in turn will reduce exposure times by at least a factor of 5. The larger photon flux will also allow serial crystallography experiments on micrometre-sized crystals. The heart of the upgrade will be the exchange of the currently used Si(111) double-crystal monochromator with a double multilayer mirror monochromator (DMM). This DMM will be optimized for an energy of around 10.5 keV and an energy bandpass of around 90 eV. Our beamline simulation results using *Ray-UI* (Baumgärtel *et al.*, 2016 ▸) show an effective photon flux of 2.2 × 10^12^  photon s^−1^ (1% bandwidth)^−1^ at the sample position, which corresponds to a 27-fold increase relative to the current situation. The monochromator exchange is planned for 2026. In anticipation of this upgrade, a Pilatus3X6M performance model detector was installed in December 2024, which allows for up to a 100 Hz frame rate.

Future automation projects are aiming to establish a fully automated beamline operation at BL14.1 for large-scale repetitive data collections, as required *e.g.* for CFS campaigns.

In addition we are currently working on a preliminary design review for the BESSY III project (Schwarzkopf *et al.*, 2023 ▸). The current plan entails the design of two MX beamlines, one high-performance microfocus beamline with possibilities for time-resolved serial crystallography and one screening beamline. The envisaged start of BESSY III beamline commissioning is currently 2036.

### 2.5. Ancillary facilities and BioLab

Several ancillary facilities and devices are available to support HZB-MX users and in-house researchers in their projects.

#### 2.5.1. Gas pressure cell

A pressure device to incubate macromolecular crystals (Hampton Research, USA) with gasses is available for probing potential binding sites of molecules. Gasses such as Xe or Kr, but also other gasses (CO_2_, NO, O_2_*etc*.) can be diffused under high pressure into the solvent channels of the incubated protein crystals (Schiltz *et al.*, 2003 ▸) to investigate binding sites, hydro­phobic channels and other properties. In the case of using Xe or Kr, heavy-atom derivatives of proteins can be prepared.

#### 2.5.2. Crystal annealing

At each beamline, crystals can be annealed for a specific time frame by a shield mounted at the nozzle of the cryo-system. This device blocks the cryo-stream for 1–10 s (Stevenson *et al.*, 2001 ▸). On all beamlines the device can be controlled remotely.

#### 2.5.3. HZB-MX BioLab

A Biosafety Level 1 laboratory (HZB-MX BioLab) is located in the vicinity of the beamlines and operated by the MX group for external users and in-house research. The laboratory is presently being expanded from an area of 70 m^2^ to 120 m^2^ and awaits an upgrade with more components (*e.g.* crystal monitoring) towards a state-of-the-art infrastructure for protein crystallization. Likewise, facilities for recombinant protein production in bacteria or yeast (Innova 43, Thermo MaxQ 6000 shakers, Cleanbench, Static Incubators), fermentation (Lambda Minifor Bioreactor), protein purification (Äkta Pure, Amersham Äkta Purifier), biophysical characterization like a real-time-PCR-based differential scanning fluorimetry setup, Tecan Spark Plate Reader and a PeqLab Nanodrop are available. The HZB BioLab offers bench space for in-house research (see below) and external users. After an online registration step, users are given access and can then conduct their experiments in the BioLab.

#### 2.5.4. HZB-MX SpectroLab

The HZB-MX SpectroLab is adjacent to the beamlines (Fig. 3 ▸) and equipped with a micro-spectrophotometer that allows measurement of the absorbance of tiny protein crystals in the UV/Vis spectral region. The illumination source is a deuterium/tungsten halogen lamp, covering the UV/Vis range from 200 to 1100 nm. The spectrometer itself is an Ocean­Insight HR2000+ES model. Protein crystals can be viewed at room temperature or cryo-cooled by an Oxford Cryojet XL. The spectrometer is operated with the *OceanView* software (https://www.oceanoptics.com). SpectroLab room lighting can be from white light or LEDs with wavelengths of 462 nm (blue), 517 nm (green), 591 nm (amber) or 629 nm (red). Dark operation is also possible. The micro-spectrophotometer can be used offline to detect color changes due to structural rearrangements or radiation damage in proteins with a color center such as a heme group or a chromophore such as retinal. Prior to data acquisition, crystals can be illuminated, spectroscopically characterized and rapidly cryo-cooled to 100 K.

#### 2.5.5. HZB-MX PrepLab

The HZB-MX PrepLab is a small laboratory adjoining the beamlines. The available equipment includes the NT8 crystallization robot (FORMULATRIX, UAE) and three stereomicroscopes (Leica, Germany) for monitoring crystal growth, as well as equipment for soaking, harvesting and crystal cryo-cooling. Sample preparation for CFS is performed in the PrepLab.

### 2.6. IT infrastructure

The operation of all motors and devices of the beamlines is controlled via *SPEC* and *TANGO* components. HZB-MX is part of the MXCuBE collaboration (Oscarsson *et al.*, 2019 ▸), and the beamline operation by users is completely handled via the *MXCuBE2* software.

Each beamline uses an individual beamline control computer and several servers to distribute various other hardware services, for example the goniometer control, sample changer *TANGO* services and detector control units. All three Pilatus3 detectors are additionally supported by Pilatus Processing Unit (PPU) servers, which offer additional CPU and storage capacities at the experimental level before the diffraction data can be transferred to the central storage facilities.

The downstream MX IT infrastructure has been optimized to ideally support experimental data collection and the processing and archiving of all data. Available storage space is continuously expanded up to a total capacity of almost 0.5 Pb. Several file and compute servers are operated, which support the main data streams from the three beamlines and the distribution of the data for the shared data analysis by the beamline users. The main network communication between the endstations and the servers is based on 10–25 Gb ethernet and storage attached fiber channel networks for rapid data transfers. On-site and remote beamline users as well as in-house researchers can make use of our optimized environment for data processing, structure solution and visualization (see also Section 2.7.1). For data processing, our pipeline developed in-house named *XDSAPP* (Sparta *et al.*, 2016 ▸) is used to automatically evaluate all datasets immediately after the completion of the data collection and to present a summary report to the users. In addition, especially for individual GUI-assisted data processing for more specialized optimization, the *XDSAPP* GUI can be used. In close collaboration with MAX IV we are hosting and using the *FragMAXapp* (Lima *et al.*, 2021 ▸) expert system for the automated parallel processing and evaluation of large screening campaigns of hundreds if not thousands of datasets. The *FragMAXapp* service runs on a dedicated 192 CPU HP-DL580 server.

#### 2.6.1. Cyber attack

On 13 June 2023, HZB became the target of a severe cyber attack. The intruders infiltrated systems and, by the night of 14–15 June had launched a large-scale encryption attack, crippling IT infrastructures across HZB. Upon discovery early on 15 June, all IT services were shut down to prevent further damage and preserve evidence. Law enforcement agencies were immediately involved, and forensic investigations began. Many systems classified as evidence could not be modified or reinstalled, significantly delaying recovery efforts. Critical systems supporting beamlines, experiments and administrative services were among those affected.

In the immediate aftermath, nearly all IT-dependent systems were inoperable. E-mail and telephony were completely unavailable, and access controls, including keys and cards for offices and experimental areas, were rendered unusable or barely functional. With central IT authentication offline, services outside HZB relying on federated logins were also inaccessible. Communication was reduced to the most basic means: in-person meetings and personal phone calls. For several weeks, these *ad hoc* methods were the only way to coordinate efforts, as no centralized communication systems were available. It took significant time to restore even minimal communication workflows, and functional systems for broader coordination were only established much later.

The full scale of the attack became apparent shortly after. Discussions on recovery began immediately, leading to the formation of the ‘BESSY II Restart’ task force within a month after the attack with the aim of overseeing the process (Müller *et al.*, 2025 ▸). For the experimental hall the recovery plan required a complete IT infrastructure overhaul, including a secure redesign of the network and dedicated infrastructure. However, with financial and administrative systems also down, procurement and payments were severely disrupted. Despite these challenges, the first shipment of new hardware arrived in September 2023, and installation began immediately. Old servers were carefully preserved for forensic analysis, while new systems were built from scratch.

By mid to late October 2023, basic configurations of servers and core infrastructure were completed, including the operation of virtualization clusters and critical backend systems. Services such as name resolution, DHCP, boot servers, file servers for the control infrastructure, proxies and jump hosts were progressively built from scratch by November. The process required meticulous documentation and automation to manage the complexity of the new setup, which isolated every experimental station with dedicated subnets and VLANs to enhance security. This work was carried out by a newly formed group, composed of personnel reassigned from various departments, as central IT resources were primarily focused on restoring foundational services. This newly assembled team was established specifically to handle the unique challenges of rebuilding the experimental hall infrastructure.

In December, prototype testing of beamlines began, refining workflows for migrating systems to the new network. The migration of approximately 50 experimental setups began in mid-January 2024 (with the total number of related services and networks amounting to approximately 140). The MX beamlines were then able to resume local operations by late January. Each migration involved identifying and documenting every network port, computer and device belonging to a particular experiment, as well as reconfiguring and testing systems and deploying all necessary services in each of the newly created networks. This phased process continued into early summer 2024.

While local beamline operations were restored by mid-2024, remote access capabilities, a critical feature particularly for MX users, were re-enabled only by October 2024 (see also Section 2.7.1 below). The priority then was to ensure secure and stable local operations before implementing advanced features. The recovery left HZB with a robust and modernized IT infrastructure, hopefully better prepared for future challenges.

### 2.7. Access to the HZB-MX beamlines

Access to the HZB-MX beamlines is organized via HZB’s digital user office portal GATE (https://www.helmholtz-berlin.de/user/gate/index_en.html). The acronym GATE stands for **G**eneral **A**ccess **T**ool to the **E**xperimental infrastructures of HZB. Within GATE all user communication is managed, from user registration to safety training, applications for beamtime, managing allocated beamtimes, including scheduling, all the way to reporting on the beamtime shifts used. MX user beamtime is typically available from Wednesday to Sunday. Monday is machine commissioning day and Tuesday is set aside for beamline commissioning. However, after successful beamline commissioning on Tuesday morning, the remaining Tuesday hours may also be used for user experiments or for in-house research.

Two calls for beamtime applications are issued per year, with deadlines typically in February or March and in September. For MX beamtime applications, user groups are requested to pool all their projects (up to 20) into a single proposal, so that they can be assessed jointly by the beamtime selection panel. In addition, upon request, MX beamtime can be applied for in a rolling or urgent access scheme throughout the year. Once submitted, the proposal will be sent out for review and beamtime can often be allocated in less than two weeks following the submission. A speciality of HZB-MX user operation is the fact that users can schedule their own beamtime using the MX calendar, which is accessible via GATE.

#### 2.7.1. Remote beamline access

Remote beamline access is enabled for BL14.1 and BL14.2. Upon booking their beamtime, beamline users can select whether they want to use this access scheme or whether they prefer to carry out their experiments in person at the HZB. After the initial establishment of remote beamline access in 2020, more than 95% of all beamtimes have been operated via the remote beamline preferred access scheme. Now, after the disruption by the cyber attack and the successful relaunch of the remote beamline operation, we anticipate returning to the previously achieved remote utilization of the beamlines in the coming months.

Technically, users connect to the relevant experimental control computers using *NoMachine* (https://www.nomachine.com) and multi-factor-authentication (MFA) access rules. Once connected, the user can access the *MXCuBE* application displayed on a dual-monitor user terminal. This enables the user to set up their data collections, and to view the robot-assisted sample transfer and the sample environment at the goniometer during the diffraction experiment via two parallel high-definition video-streams. Users can analyze the auto-processing results or can run interactive processing. The local user support is connected via *Zoom* to the remote users for training at the beginning and during normal working hours of the experiment. HZB-MX supports a 24/7 call service for local technical support, which can be called by the users in case of problems at any time.

Several times a year a remote access online training is organized by HZB-MX, which all remote users are obliged to attend at least once per year. During this 3–4 h training, the experimental capabilities are discussed as well as all required steps to prepare and execute remote experiments at the beamlines.

## 3. Scientific output

**Figure 6:**
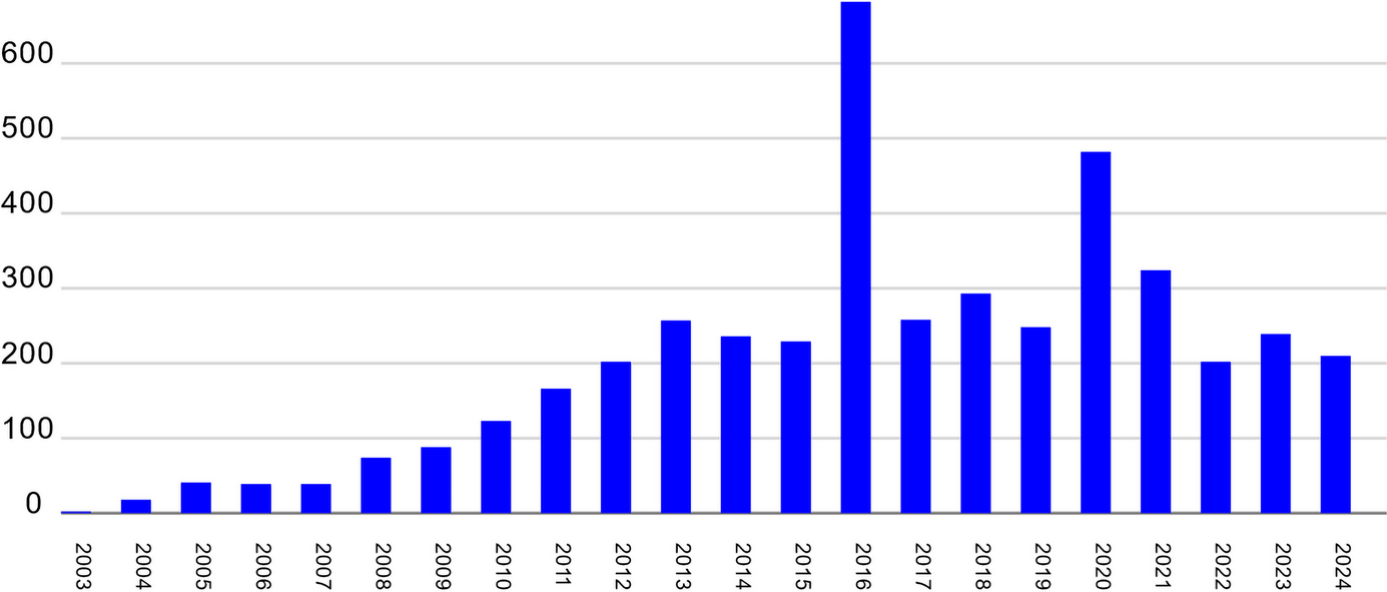
Yearly PDB deposition statistics of the HZB-MX beamlines (status 10 December 2024).

Since the first structure based on data collected on the HZB-MX beamlines was published in 2003, more than 4500 further structures have been deposited in the PDB. The number of structures deposited in the PDB per year is shown in Fig. 6 ▸. After the initial ramp-up phase 2003–2009, the output is now relatively stable at 200–300 structures per year. In addition to macromolecular structures, several hundred small-molecule structures have been determined based on data collected at the beamlines. With respect to publications, the last reliable numbers stem from the period 2012–2016 due to a beamline review, which took place in early 2017. In this period 705 scientific publications, 184 completed PhD theses and 85 Diploma/Master theses were based in full or in part on data collected at one of the HZB-MX beamlines. Compared with the previous period 2007–2011, this represented essentially a doubling of the numbers.

## 4. In-house research program

### 4.1. Synchrotron-based fragment screening

After the installation of the first Pilatus2 detector in early 2013 on BL14.1, it was realized that the sample throughput of the beamline could be increased to the extent that screening experiments could become feasible. Consequently, activities were initiated to establish a CFS facility. In collaboration with the drug design group of Professor Dr Gerhard Klebe, University of Marburg, Germany, and supported by two grants from the German Research Ministry BMBF, the two compound screens F2X-Entry and F2X-Universal (Wollenhaupt *et al.*, 2020 ▸) and tools to support the handling of large sample numbers were developed (Barthel *et al.*, 2021 ▸), and a complete CFS workflow was established (Wollenhaupt *et al.*, 2021 ▸). The facility, called the F2X facility (Barthel *et al.*, 2024 ▸), is now fully operational (https://hz-b.de/F2X). It provides users and in-house researchers with the possibility of screening their own target protein(s) for potential starting points in a drug discovery project.

### 4.2. Plastic-degrading enzyme design

**Figure 7:**
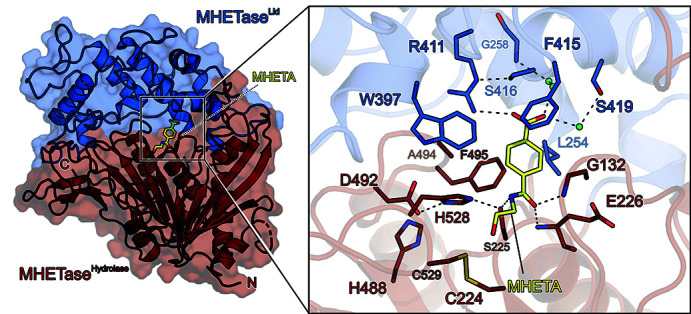
The structure of *I. sakaiensis* MHETase bound to the non-hydrolyzable substrate MHETA (PDB ID 6qga). An overview of MHETase shown as a cartoon with a transparent surface. The hydro­lase domain is colored in firebrick, the lid domain in marine and MHETA as yellow sticks. On the right close-up view of the MHETase catalytic triad, oxyanion hole, substrate-binding residues and the water molecules in the active site cavity. Dashed lines indicate hydrogen bonds, interacting residues are shown as sticks and colored by atom type. Carbon, as given for the respective molecule; nitro­gen, blue; oxygen, red; sulfur, yellow. Water oxygens are shown as light green spheres.

In 2016, the discovery of the bacterium *I. sakaiensis* which naturally evolved two enzymes, coined PETase [breaking down polyethyl­ene terephthalate (PET) to the intermediate mono-hy­droxy ethyl­ene terephthalate, MHET] and MHETase (hydrolyzing MHET to the monomeric building blocks terephthalic acid and ethyl­ene glycol) (Bornscheuer, 2016 ▸; Yoshida *et al.*, 2016 ▸), sparked great interest in enzymatic plastic degradation. In recent years, enzymes have become a viable option for the recycling, upcycling and bioremediation of hydrolyzable polymer types, *i.e.* PET, PU (polyurethane) or PA (polyamide). The project started with the first structures of *I. sakaiensis* PETase and MHETase (Graf *et al.*, 2021 ▸; Palm *et al.*, 2019 ▸). However, due to their low activity and low stability, these enzymes need further improvement before they qualify for more sustainable larger-scale industrial recycling processes. In particular, the structure of substrate-bound MHETase (Fig. 7 ▸) was instrumental in improving the enzyme activity semi-rationally by a factor of two and even led to the design of a new enzyme that can hydrolyze the related substrate bis-hy­droxy­ethyl­ene terephthalate (BHET). Together with collaborators, multiple other PET hydro­lases were structurally characterized and improved (Wei *et al.*, 2022 ▸; Pfaff *et al.*, 2022 ▸). In these studies, carboxyl­esterases were structurally examined, and alternative substrate-binding modes of PET hydro­lases were elucidated by co-crystal structures. To improve the comparability and coherence of new studies on PET hydro­lases, standardized experimental parameters for the assessment of PET hydro­lases were suggested together with academic and industrial collaborators (Arnal *et al.*, 2023 ▸). Ongoing studies on PET hydrolysis have currently expanded towards other polymer types like PA and PU, also implementing AI-based enzyme design (Dauparas *et al.*, 2022 ▸; Wang *et al.*, 2022 ▸; Krishna *et al.*, 2024 ▸). Recently, it was possible to structurally characterize a polyurethanase, which possesses an amidase fold along with an unusual regulation mechanism (Li *et al.*, 2025 ▸). The crystal structures further served as a starting point for quantum mechanics/molecular mechanics analyses to elucidate the enzyme’s intricate catalytic cycle (Paiva *et al.*, 2025 ▸). Additionally, nylonases Nyl-C and Nyl-A will be further investigated structurally to improve the active site accessibility either rationally or with AI-based enzyme design (Yasuhira *et al.*, 2010 ▸; Bell *et al.*, 2024 ▸).

## 5. Beamline user research highlights

### 5.1. SARS-CoV-2 main protease (Hilgenfeld group, Lübeck University)

**Figure 8:**
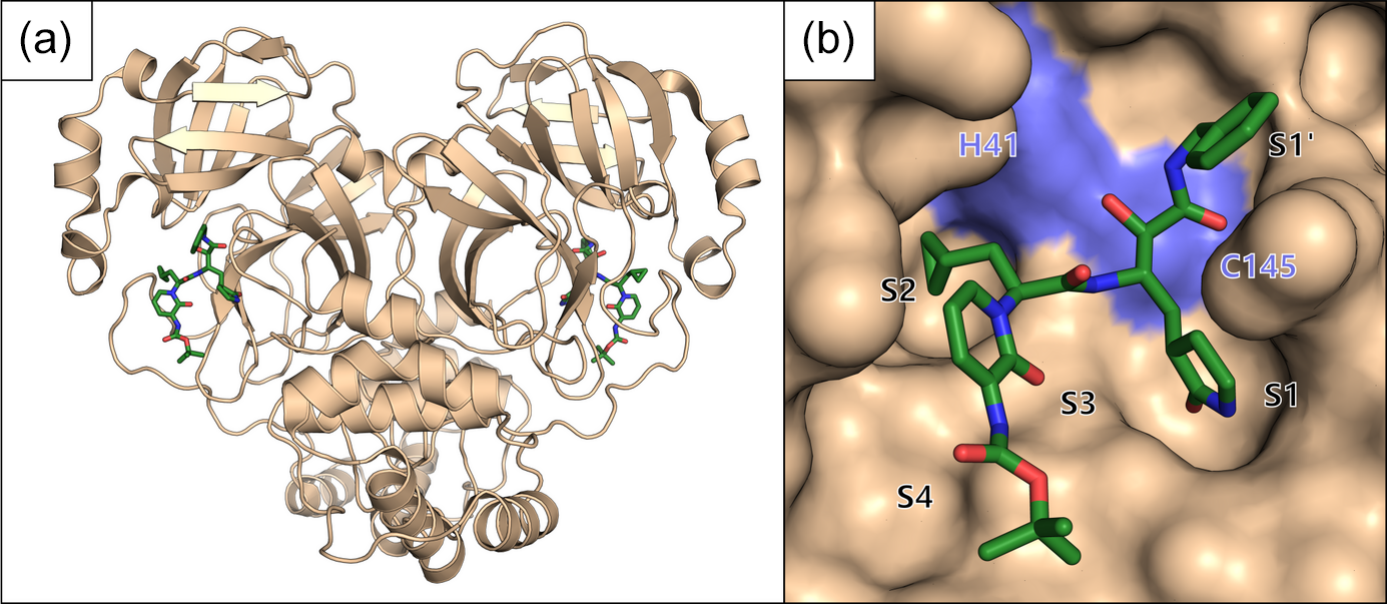
The crystal structure of MPro from the Hilgenfeld group (PDB ID 6y2g). (*a*) A cartoon overview of the dimer of MPro shown in beige with the bound ligand in stick representation, colored in green. (*b*) A detailed view of the active site of MPro with the protein’s surface shown in beige, except for the two active site residues shown in blue. The ligand 13b is shown as sticks colored in green.

In 2020, the Covid-19 pandemic appeared and shortly after turned into a global health emergency. The first incidents of a newly emerging respiratory disease became public in December 2019, and the source of this new disease was quickly identified as a novel coronavirus in January 2020. Only days after the viral genome was published the Hilgenfeld group began to produce the main protease Mpro of the new virus (later called SARS-CoV-2). After successful crystallization, they reached out to the MX group at BESSY II for beamtime, and three days later managed to collect a high-resolution dataset (1.75 Å) at BL14.2 of their Mpro crystals. The structure was then solved in early February 2020 (Fig. 8 ▸) and published in March 2020 (Zhang *et al.*, 2020 ▸). These studies led to the first structural insights into the newly emerged virus, which were used as a basis for several other experiments and hypotheses.

### 5.2. Phytochrome (Hughes group, Giessen University)

**Figure 9:**
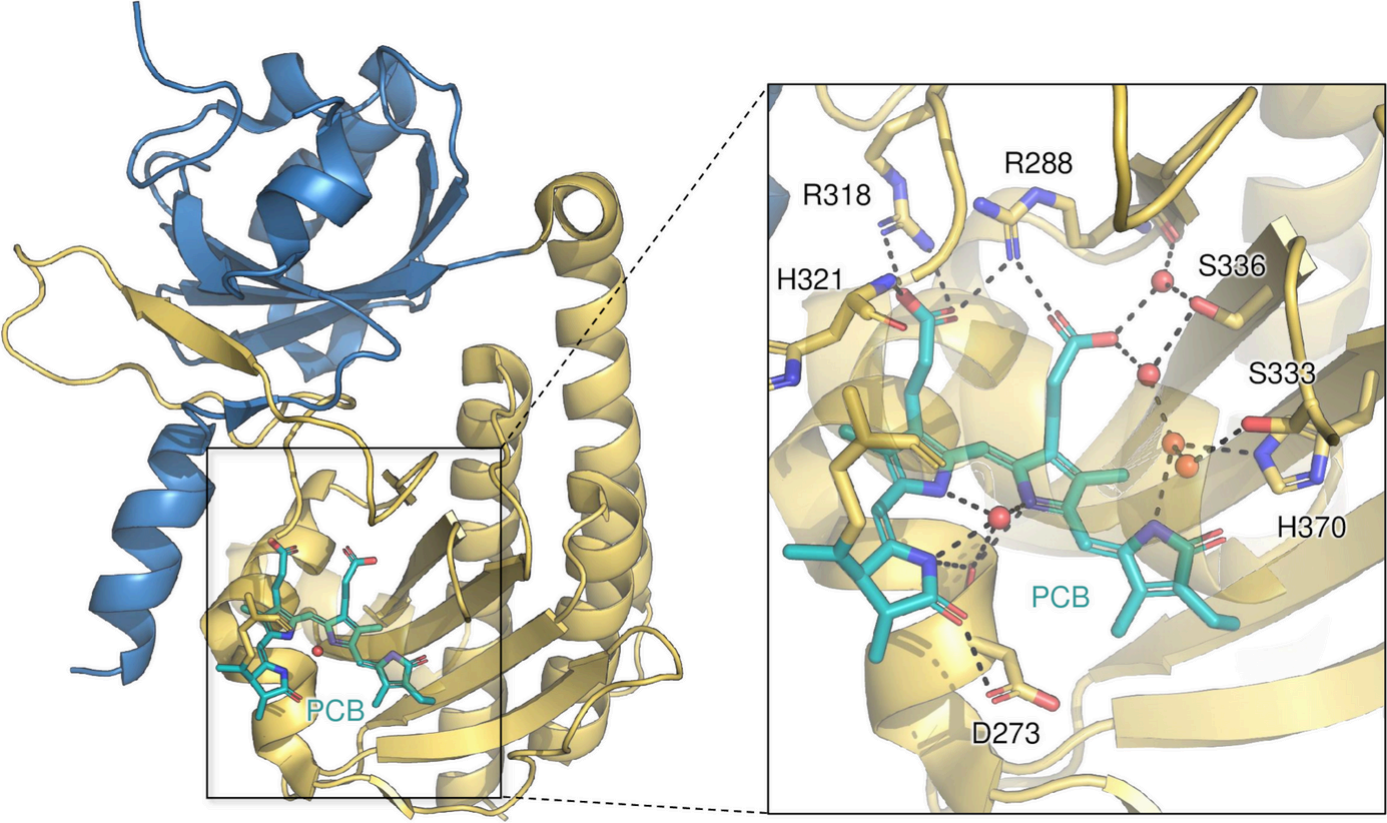
Structure of soybean PhyA(PG)-PCB. The PCB chromophore is colored in cyan, and the PAS and GAF subunits of PhyA are shown in blue and yellow, respectively. Waters are shown as red spheres. The inset shows the details of the hydrogen bonding (black lines) network with PCB.

Phytochromes are a class of photoreceptors that are used by prokaryotes and plants to sense their environment and regulate key developmental processes. They exist in a red-light sensing Pr and a far-red-light sensing Pfr state, between which they can photoconvert through absorption of a photon at their bilin chromophore. In plants, different groups of phytochromes predominate under different environmental conditions, for example phyA in the dark or phyB under light conditions. To understand the structure and function of these different phytochromes, crystals of the sensory modules of soybean and sorghum phytochromes in the Pr state were measured at BL14.1. This yielded structures at high resolutions (PDB IDs 6tl4, 6tc5 and 6tc7), including the first structure of a plant phyA phytochrome (Nagano *et al.*, 2020 ▸). Two of these datasets have been collected at BL14.2 at maximum resolutions ranging from 2.1 to 2.9 Å. These structures reveal details of how the chromophore is bound (Fig. 9 ▸) and, together with spectroscopic measurements, suggest mechanisms of how plant phytochromes convert between the Pr and Pfr states, further highlighting intriguing differences as well as similarities between phytochromes from plants and prokaryotes.

### 5.3. PI3KC2α core in complex with PITCOIN2 (Daumke group, Max Delbrück Centrum Berlin-Buch)

**Figure 10:**
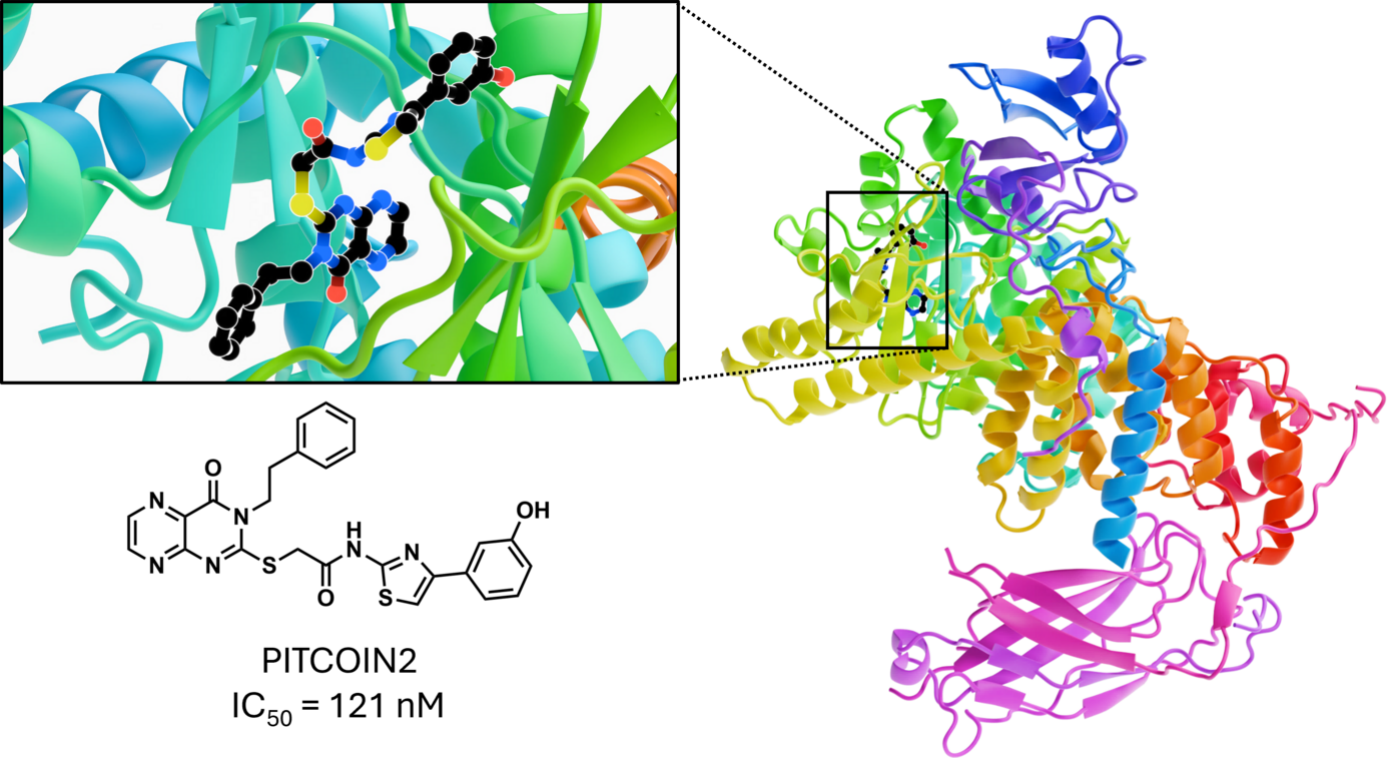
PITCOIN2 in complex with PI3KC2α taken from PDB ID 7z74 (Lo *et al.*, 2023 ▸). The binding site is magnified and shown in more detail. The structure of PITCOIN2 is also shown along with the IC50 value determined experimentally.

Phosphatidylinositol 3-kinase type 2α (PI3KC2α) is a member of the lipid-modifying PI3K family. Due to its roles in cancer, diabetes and neurological disorders, it has significant medical relevance in humans. PI3KC2α is a class II PI3K, and it synthesizes PI3,4-bis­phosphate [PI(3,4)P_2_] from PI 4-phosphate at the plasma membrane, and is essential in mice. While PI3KC2α performs important cellular functions, it is also implicated in cancer, cataract formation, viral replication, and other morbidities. Hence there is a demand for inhibitors that target specific PI3K enzymes for therapeutic treatment. Using data collected at HZB-MX BL14.1, structures of PI3KC2α (Fig. 10 ▸) bound to three **P**hosphatidyl**I**nositol **T**hree-kinase **C**lass tw**O** I**N**hibitors (PITCOINs) were solved to resolutions of 2.5–2.9 Å (PDB IDs 8a9i, 7z74, 7z75) (Lo *et al.*, 2023 ▸). PITCOIN2 displayed an IC50 value of 121 n*M* with a high specificity and the least off-target effects due to extra hydro­phobic solvent-mediated stabilization with its inward-facing hy­droxy­phenyl group. In follow-up experiments, it was found that PITCOIN1 and 3 impair PI(3,4)P_2_ synthesis while displaying no cytotoxicity, suggesting they are suitable candidates for further drug development.

### 5.4. Metal–Organic Frameworks (Kaskel group, Dresden University of Technology)

**Figure 11:**
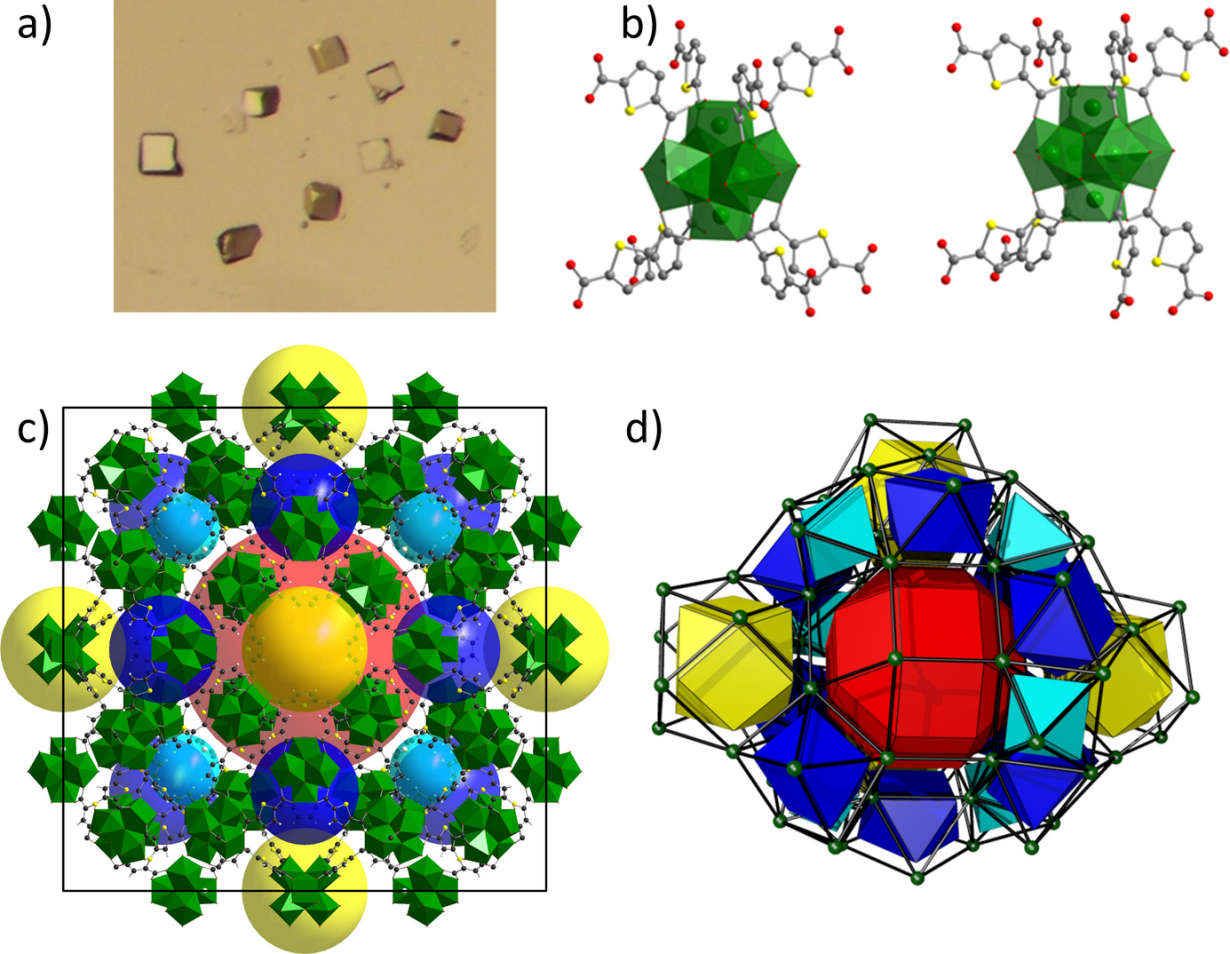
Illustration of the single crystals (*a*), secondary building units (*b*), crystal structure (*c*) and topological representation (*d*) of the Zr-based mesoporous framework DUT-68(Zr) [pores are shown as spheres (*c*) and geometric shapes (*d*)].

Metal–Organic Frameworks (MOFs) are an emerging class of porous crystalline solids constructed from metal clusters connected into 3D frameworks by organic ligands, adhering to a modular building principle (Eddaoudi *et al.*, 2001 ▸). They hold record-breaking values in specific surface area and pore volume, making them suitable for a range of applications, including gas storage, separation, catalysis, drug delivery, sensors and actuators (Kaskel, 2016 ▸). Depending on synthesis conditions, MOFs can crystallize as either single crystals (10–500 µm) or powders (10–1000 nm) (Schaate *et al.*, 2011 ▸). Even when single crystals are obtained, determining their crystal structure is challenging due to the high content (up to 90%) of disordered guest molecules, typically organic solvents, within the pores. The ordered portion of the crystal structure usually represents only 10–50% of the crystal volume, and the unit cells can be substantial, often exceeding 100000 Å^3^ in volume. This complexity poses challenges for structure determination using laboratory single-crystal X-ray diffractometers, even those equipped with microfocus X-ray sources. Due to similarities between MOF and protein crystals, researchers of the Kaskel group at TU Dresden frequently collect data at the HZB-MX beamlines. Since 2009 the crystal structures of nearly 80 new crystalline frameworks have been solved, refined and published from datasets collected at beamlines BL14.2 and BL14.3. In some cases, multiple datasets were required either collected at different temperatures or collected on crystals containing different guest molecules in the pores. As a result, more than 130 new MOF-structures have been added to the Cambridge Structural Database, and 35 papers have been published in high-impact journals. For example, the crystal structures of ultra high porosity frameworks with surface areas greater than 5000 m^2^ g^−1^ have been solved and refined from datasets collected at BL14.2 (Klein *et al.*, 2009 ▸; Grünker *et al.*, 2014 ▸; Grünker *et al.*, 2012 ▸; Stoeck *et al.*, 2012 ▸; Krause *et al.*, 2019 ▸; Ehrling *et al.*, 2021 ▸; Garai *et al.*, 2020*a* ▸; Garai *et al.*, 2020*b* ▸; Müller *et al.*, 2015 ▸). A typical example of the single crystals and crystal structure of a mesoporous Zr-based framework is depicted in Fig. 11 ▸. The dataset, collected at BL14.2, allows one to solve and refine the crystal structure of DUT-68(Zr) (DUT – Dresden University of Technology. It crystallizes in a cubic space group 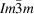 [*a* = 53.680 (6) Å, *V* = 154681 (31) Å^3^]. Although constructed of relatively small building blocks [Fig. 11 ▸(*b*)], the crystal structure shows a complex framework topology, resulting in the formation of a hierarchical pore structure containing the rhombicuboctahedral mesopore of 28 Å in diameter [Figs. 11 ▸(*c*), 11 ▸(*d*)] (Bon *et al.*, 2013 ▸).

## 6. Summary and outlook

In this contribution, we have presented the history and status of the three MX beamlines operated at BESSY II in Berlin, Germany. Even though the beamlines have been in operation since 2003, they continue to serve a large and loyal user community, and they still belong to the most productive installations of their kind in Europe. With the imminent upgrade on BL14.1 to a pink beamline, the need for higher sample throughput in particular for CFS experiments will be met. Similarly, the HZB-MX group will continue to work towards keeping the beamlines state-of-the-art and competitive on an international level.

## Figures and Tables

**Table 1:** Summary of beamline properties

	BL14.1	BL14.2	BL14.3
Wavelength range (Å)	0.8–2.25	0.8–2.25	0.89
Photon flux at sample [photon s^−1^ (100 mA)^−1^ (0.05% bandwidth)^−1^]	1.6 × 10^11^ (at λ = 0.92 Å)	1.5 × 10^11^ (at λ = 0.92 Å)	2.3 × 10^10^
Energy resolution (eV)	< 2	< 2	< 5
Beam size (µm diameter)	50–100	100	50–200
Goniometer	MD2 microdiffractometer with MK3	Nanodiffractometer	MD2S microdiffractometer with MK3
X-ray detector	Pilatus3 X 6M	Pilatus3 S 2M	Pilatus3 S 6M
Sample mounting	CATS sample changer	ISARA2 sample changer	Manual
No. of samples in sample dewar	144 Unipuck	464 Unipuck	–
Exposure times (s °^−1^)	0.1–10	0.4–10	0.4–20
Detector distance range (mm)	129–649	57–800	110–501
Achievable resolution (Å)	0.84	0.71	0.85
Maximum unit-cell length (Å)	600 (at *d*_min_ = 2.0 Å)	400 (at *d*_min_ = 2.0 Å)	600 (at *d*_min_ = 2.0 Å)
Remote operation	Yes	Yes	No
Special equipment and operations	HT crystal screening	HT crystal screening	RT data collection
	Crystal annealing	Safe light conditions	Controlled dehydration
	UV-pulsed laser for ultra-violet light radiation-damage inducing phasing	Chemical crystallography	REX nozzle exchanger

## References

[bb1] Arnal, G., Anglade, J., Gavalda, S., Tournier, V., Chabot, N., Bornscheuer, U. T., Weber, G. & Marty, A. (2023). *ACS Catal.* **13**, 13156–13166. 10.1021/acscatal.3c02922 PMC10594578 37881793

[bb3] Barthel, T., Benz, L., Basler, Y., Crosskey, T., Dillmann, A., Förster, R., Fröling, P., Dieguez, C. G., Gless, C., Hauß, T., Hellmig, M., Jänisch, L., James, D., Lennartz, F., Mijatovic, J., Oelker, M., Scanlan, J. W., Weber, G., Wollenhaupt, J., Mueller, U., Dobbek, H., Wahl, M. C. & Weiss, M. S. (2024). *Appl. Res.* **3**, e202400110.

[bb4] Barthel, T., Huschmann, F. U., Wallacher, D., Feiler, C. G., Klebe, G., Weiss, M. S. & Wollenhaupt, J. (2021). *J. Appl. Cryst.* **54**, 376–382. 10.1107/S1600576720016477 PMC7941301 33833659

[bb5] Baumgärtel, P., Witt, M., Baensch, J., Fabarius, M., Erko, A., Schäfers, F. & Schirmacher, H. (2016). *AIP Conf. Proc.* **1741**, 040016.

[bb6] Bell, E. L., Rosetto, G., Ingraham, M. A., Ramirez, K. J., Lincoln, C., Clarke, R. W., Gado, J. E., Lilly, J. L., Kucharzyk, K. H., Erickson, E. & Beckham, G. T. (2024). *Nat. Commun.* **15**, 1217. 10.1038/s41467-024-45523-5 PMC10858056 38336849

[bb7] Berman, H. M., Westbrook, J., Feng, Z., Gilliland, G., Bhat, T. N., Weissig, H., Shindyalov, I. N. & Bourne, P. E. (2000). *Nucleic Acids Res.* **28**, 235–242. 10.1093/nar/28.1.235 PMC102472 10592235

[bb8] Bon, V., Senkovska, I., Baburin, I. A. & Kaskel, S. (2013). *Cryst. Growth Des.* **13**, 1231–1237.

[bb9] Bornscheuer, U. T. (2016). *Science*, **351**, 1154–1155. 10.1126/science.aaf2853 26965614

[bb101] Bowler, M. W., Mueller, U., Weiss, M. S., Sanchez-Weatherby, J., Sorensen, T. L. M., Thunnissen, M. M. G. M., Ursby, T., Gobbo, A., Russi, S., Bowler, M. G., Brockhauser, S., Svensson, O. & Cipriani, F. (2015). *Cryst. Growth Des.* **15**, 1043–1054.

[bb10] Dauparas, J., Anishchenko, I., Bennett, N., Bai, H., Ragotte, R. J., Milles, L. F., Wicky, B. I. M., Courbet, A., de Haas, R. J., Bethel, N., Leung, P. J. Y., Huddy, T. F., Pellock, S., Tischer, D., Chan, F., Koepnick, B., Nguyen, H., Kang, A., Sankaran, B., Bera, A. K., King, N. P. & Baker, D. (2022). *Science*, **378**, 49–56. 10.1126/science.add2187 PMC9997061 36108050

[bb11] Eddaoudi, M., Moler, D. B., Li, H., Chen, B., Reineke, T. M., O’Keeffe, M. & Yaghi, O. M. (2001). *Acc. Chem. Res.* **34**, 319–330. 10.1021/ar000034b 11308306

[bb12] Ehrling, S., Reynolds, E. M., Bon, V., Senkovska, I., Gorelik, T. E., Evans, J. D., Rauche, M., Mendt, M., Weiss, M. S., Pöppl, A., Brunner, E., Kaiser, U., Goodwin, A. L. & Kaskel, S. (2021). *Nat. Chem.* **13**, 568–574. 10.1038/s41557-021-00684-4 34045713

[bb13] Fearon, D., Powell, A., Douangamath, A., Dias, A., Tomlinson, C. W. E., Balcomb, B. H., Aschenbrenner, J. C., Aimon, A., Barker, I. A., Brandão–Neto, J., Collins, P., Dunnett, L. E., Fairhead, M., Gildea, R. J., Golding, M., Gorrie–Stone, T., Hathaway, P. V., Koekemoer, L., Krojer, T., Lithgo, R. M., Maclean, E. M., Marples, P. G., Ni, X., Skyner, R., Talon, R., Thompson, W., Wild, C. F., Winokan, M., Wright, N. D., Winter, G., Shotton, E. J., von Delft, F., Bertram, F., Coe, P. A., Mikolajek, H., Nidamarthi, K. H. V., O’Donnell, G., Watt, G. & Williams, M. A. (2025). *Appl. Res.* **4**, e202400192.

[bb14] Fraser, J. S., van den Bedem, H., Samelson, A. J., Lang, P. T., Holton, J. M., Echols, N. & Alber, T. (2011). *Proc. Natl Acad. Sci. USA*, **108**, 16247–16252. 10.1073/pnas.1111325108 PMC3182744 21918110

[bb15] Garai, B., Bon, V., Efimova, A., Gerlach, M., Senkovska, I. & Kaskel, S. (2020*a*). *J. Mater. Chem. A*, **8**, 20420–20428.

[bb16] Garai, B., Bon, V., Krause, S., Schwotzer, F., Gerlach, M., Senkovska, I. & Kaskel, S. (2020*b*). *Chem. Mater.* **32**, 889–896. 10.1021/acs.chemmater.9b04769 PMC9115755 35601600

[bb17] Graf, L. G., Michels, E. A. P., Yew, Y., Liu, W., Palm, G. J. & Weber, G. (2021). *Methods Enzymol.* **648**, 337–356. 10.1016/bs.mie.2020.12.015 33579411

[bb18] Grünker, R., Bon, V., Heerwig, A., Klein, N., Müller, P., Stoeck, U., Baburin, I. A., Mueller, U., Senkovska, I. & Kaskel, S. (2012). *Chem. A Eur. J.* **18**, 13299–13303. 10.1002/chem.201202352 22976851

[bb19] Grünker, R., Bon, V., Müller, P., Stoeck, U., Krause, S., Mueller, U., Senkovska, I. & Kaskel, S. (2014). *Chem. Commun.* **50**, 3450–3452. 10.1039/c4cc00113c 24549108

[bb20] Heinemann, U. (2000). *Nat. Struct. Biol.* **7**, 940–942. 10.1038/80707 11103993

[bb21] Heinemann, U., Büssow, K., Mueller, U. & Umbach, P. (2003). *Acc. Chem. Res.* **36**, 157–163. 10.1021/ar010129t 12641472

[bb22] Heinemann, U., Frevert, J., Hofmann, K.-P., Illing, G., Maurer, C., Oschkinat, H. & Saenger, W. (2000). *Prog. Biophys. Mol. Biol.* **73**, 347–362. 10.1016/s0079-6107(00)00009-2 11063780

[bb23] Jumper, J., Evans, R., Pritzel, A., Green, T., Figurnov, M., Ronneberger, O., Tunyasuvunakool, K., Bates, R., Žídek, A., Potapenko, A., Bridgland, A., Meyer, C., Kohl, S. A. A., Ballard, A. J., Cowie, A., Romera-Paredes, B., Nikolov, S., Jain, R., Adler, J., Back, T., Petersen, S., Reiman, D., Clancy, E., Zielinski, M., Steinegger, M., Pacholska, M., Berghammer, T., Bodenstein, S., Silver, D., Vinyals, O., Senior, A. W., Kavukcuoglu, K., Kohli, P. & Hassabis, D. (2021). *Nature*, **596**, 583–589. 10.1038/s41586-021-03819-2 PMC8371605 34265844

[bb24] Kanchugal, P. S., Jagudin, E., Lima, G. M. A., Talibov, V. O., Begum, A., Nan, J., Eguiraun, M., Gonzalez, A., Sele, C., Nyblom, M., Knecht, W., Logan, D. T., Sjögren, T., Thunnissen, M., Ursby, T., Obiols–Rabasa, M., Larsson, M., Mueller, U. & Krojer, T. (2025). *Appl. Res.* **4**, e202400263.

[bb25] Kaskel, S. (2016). Editor. *The Chemistry of Metal-Organic Frameworks: Synthesis, Characterization, and Applications.* Weinheim: Wiley-VCH.

[bb26] Kiefersauer, R., Than, M. E., Dobbek, H., Gremer, L., Melero, M., Strobl, S., Dias, J. M., Soulimane, T. & Huber, R. (2000). *J. Appl. Cryst.* **33**, 1223–1230.

[bb27] Kigawa, T., Yabuki, T., Shirouzu, M., Terada, T., Ito, Y., Matsuo, Y., Kuroda, Y., Nishimura, Y., Kyogoku, Y., Miki, K., Masui, R. & Kuramitsu, S. (2000). *Nat. Struct. Biol.* **7**, 943–945. 10.1038/80712 11103994

[bb28] Klein, N., Senkovska, I., Gedrich, K., Stoeck, U., Henschel, A., Mueller, U. & Kaskel, S. (2009). *Angew. Chem. Int. Ed.* **48**, 9954–9957. 10.1002/anie.200904599 19937878

[bb29] Klingl, S., Scherer, M., Stamminger, T. & Muller, Y. A. (2015). *Acta Cryst.* D**71**, 1493–1504. 10.1107/S1399004715008792 26143921

[bb30] Krause, S., Evans, J. D., Bon, V., Senkovska, I., Iacomi, P., Kolbe, F., Ehrling, S., Troschke, E., Getzschmann, J., Többens, D. M., Franz, A., Wallacher, D., Yot, P. G., Maurin, G., Brunner, E., Llewellyn, P. L., Coudert, F. & Kaskel, S. (2019). *Nat. Commun.* **10**, 3632. 10.1038/s41467-019-11565-3 PMC6690989 31406113

[bb31] Krishna, R., Wang, J., Ahern, W., Sturmfels, P., Venkatesh, P., Kalvet, I., Lee, G. R., Morey-Burrows, F. S., Anishchenko, I., Humphreys, I. R., McHugh, R., Vafeados, D., Li, X., Sutherland, G. A., Hitchcock, A., Hunter, C. N., Kang, A., Brackenbrough, E., Bera, A. K., Baek, M., DiMaio, F. & Baker, D. (2024). *Science*, **384**, eadl2528.10.1126/science.adl252838452047

[bb32] Kühlbrandt, W. (2014). *Science*, **343**, 1443–1444. 10.1126/science.1251652 24675944

[bb102] Li, Z., Han, X., Cong, L., Singh, P., Paiva, P., Branson, Y., Li, W., Chen, Y., Jaradat, D. M. M., Lennartz, F., Bayer, T., Schmidt, L., Garscha, U., You, S., Fernandes, P. A., Ramos, M. J., Bornscheuer, U. T., Weber, G., Wei, R. & Liu, W. (2025). *Adv. Sci.* **2025**, 2416019 10.1002/advs.202416019 PMC11967865 39921299

[bb33] Lima, G. M. A., Jagudin, E., Talibov, V. O., Benz, L. S., Marullo, C., Barthel, T., Wollenhaupt, J., Weiss, M. S. & Mueller, U. (2021). *Acta Cryst.* D**77**, 799–808. 10.1107/S2059798321003818 PMC8171072 34076593

[bb34] Lo, W. T., Belabed, H., Kücükdisli, M., Metag, J., Roske, Y., Prokofeva, P., Ohashi, Y., Horatscheck, A., Cirillo, D., Krauss, M., Schmied, C., Neuenschwander, M., von Kries, J. P., Médard, G., Kuster, B., Perisic, O., Williams, R. L., Daumke, O., Payrastre, B., Severin, S., Nazaré, M. & Haucke, V. (2023). *Nat. Chem. Biol.* **19**, 18–27. 10.1038/s41589-022-01118-z PMC7613998 36109648

[bb35] Malinauskaite, L., Quick, M., Reinhard, L., Lyons, J. A., Yano, H., Javitch, J. A. & Nissen, P. (2014). *Nat. Struct. Mol. Biol.* **21**, 1006–1012. 10.1038/nsmb.2894 PMC4346222 25282149

[bb36] Monecke, T., Dickmanns, A., Weiss, M. S., Port, S. A., Kehlenbach, R. H. & Ficner, R. (2015). *Acta Cryst.* F**71**, 1481–1487. 10.1107/S2053230X15021524 PMC4666476 26625290

[bb37] Mueller, U., Darowski, N., Fuchs, M. R., Förster, R., Hellmig, M., Paithankar, K. S., Pühringer, S., Steffien, M., Zocher, G. & Weiss, M. S. (2012). *J. Synchrotron Rad.* **19**, 442–449. 10.1107/S0909049512006395 PMC3408958 22514183

[bb38] Mueller, U., Förster, R., Hellmig, M., Huschmann, F., Kastner, A., Malecki, P., Pühringer, S., Röwer, M., Sparta, K., Steffien, M., Ühlein, M., Wilk, P. & Weiss, M. S. (2015). *Eur. Phys. J. Plus*, **130**, 141–150.

[bb39] Müller, P., Wisser, F. M., Bon, V., Grünker, R., Senkovska, I. & Kaskel, S. (2015). *Chem. Mater.* **27**, 2460–2467.

[bb40] Müller, R., Meier, M., Graboswski, B., Herrendörfer, D., Ovsyannikov, R., Schälicke, A., Viefhaus, J. & Jankowiak, A. (2025). *J. Phys. Conf. Ser.* To be published.

[bb41] Nagano, S., Guan, K., Shenkutie, S. M., Feiler, C., Weiss, M., Kraskov, A., Buhrke, D., Hildebrandt, P. & Hughes, J. (2020). *Nat. Plants*, **6**, 581–588. 10.1038/s41477-020-0638-y 32366982

[bb42] Oscarsson, M., Beteva, A., Flot, D., Gordon, E., Guijarro, M., Leonard, G., McSweeney, S., Monaco, S., Mueller-Dieckmann, C., Nanao, M., Nurizzo, D., Popov, A., von Stetten, D., Svensson, O., Rey-Bakaikoa, V., Chado, I., Chavas, L., Gadea, L., Gourhant, P., Isabet, T., Legrand, P., Savko, M., Sirigu, S., Shepard, W., Thompson, A., Mueller, U., Nan, J., Eguiraun, M., Bolmsten, F., Nardella, A., Milàn-Otero, A., Thunnissen, M., Hellmig, M., Kastner, A., Schmuckermaier, L., Gerlach, M., Feiler, C., Weiss, M. S., Bowler, M. W., Gobbo, A., Papp, G., Sinoir, J., McCarthy, A., Karpics, I., Nikolova, M., Bourenkov, G., Schneider, T., Andreu, J., Cuní, G., Juanhuix, J., Boer, R., Fogh, R., Keller, P., Flensburg, C., Paciorek, W., Vonrhein, C., Bricogne, G. & de Sanctis, D. (2019). *J. Synchrotron Rad.* **26**, 393–405. 10.1107/S1600577519001267 PMC6412183 30855248

[bb43] Paiva, P., Teixeira, L. M. C., Wei, R., Liu, W., Weber, G., Morth, J. P., Westh, P., Petersen, A. R., Johansen, M. B., Sommerfeldt, A., Sandahl, A., Otzen, D. E., Fernandes, P. A. & Ramos, M. J. (2025). *Chem. Sci.* **16**, 2437–2452. 10.1039/d4sc06688j PMC11708778 39790984

[bb44] Palm, G. J., Reisky, L., Böttcher, D., Müller, H., Michels, E. A. P. P., Walczak, M. C., Berndt, L., Weiss, M. S., Bornscheuer, U. T. & Weber, G. (2019). *Nat. Commun.* **10**, 1717. 10.1038/s41467-019-09326-3 PMC6461665 30979881

[bb45] Pfaff, L., Gao, J., Li, Z., Jäckering, A., Weber, G., Mican, J., Chen, Y., Dong, W., Han, X., Feiler, C. G., Ao, Y. F., Badenhorst, C. P. S., Bednar, D., Palm, G. J., Lammers, M., Damborsky, J., Strodel, B., Liu, W., Bornscheuer, U. T. & Wei, R. (2022). *ACS Catal.* **12**, 9790–9800. 10.1021/acscatal.2c02275 PMC9361285 35966606

[bb46] Schaate, A., Roy, P., Godt, A., Lippke, J., Waltz, F., Wiebcke, M. & Behrens, P. (2011). *Chem. A Eur. J.* **17**, 6643–6651. 10.1002/chem.201003211 21547962

[bb47] Schiltz, M., Fourme, R. & Prangé, T. (2003). *Methods Enzymol.* **374**, 83–119. 10.1016/S0076-6879(03)74004-X 14696369

[bb48] Schwarzkopf, O., Jankowiak, A., Vollmer, A. & BESSYII/BESSYIII Team, (2023). *Eur. Phys. J. Plus*, **138**, 348. 10.1140/epjp/s13360-023-03957-8 PMC10119534 37124344

[bb49] Sparta, K. M., Krug, M., Heinemann, U., Mueller, U. & Weiss, M. S. (2016). *J. Appl. Cryst.* **49**, 1085–1092.

[bb51] Stevenson, C. E. M., Mayer, S. M., Delarbre, L. & Lawson, D. M. (2001). *J. Cryst. Growth*, **232**, 629–637.

[bb52] Stoeck, U., Krause, S., Bon, V., Senkovska, I. & Kaskel, S. (2012). *Chem. Commun.* **48**, 10841–10843. 10.1039/c2cc34840c 23033252

[bb53] Terwilliger, T. C. (2000). *Nat. Struct. Biol.* **7**, 935–939. 10.1038/80700 11103992

[bb54] Terwilliger, T. C., Liebschner, D., Croll, T. I., Williams, C. J., McCoy, A. J., Poon, B. K., Afonine, P. V., Oeffner, R. D., Richardson, J. S., Read, R. J. & Adams, P. D. (2024). *Nat. Methods*, **21**, 110–116. 10.1038/s41592-023-02087-4 PMC10776388 38036854

[bb60] Vitkup, D., Melamud, E., Moult, J. & Sander, C. (2001). *Nat. Struct. Biol.* **8**, 559–566. 10.1038/88640 11373627

[bb55] Wang, J., Lisanza, S., Juergens, D., Tischer, D., Watson, J. L., Castro, K. M., Ragotte, R., Saragovi, A., Milles, L. F., Baek, M., Anishchenko, I., Yang, W., Hicks, D. R., Expòsit, M., Schlichthaerle, T., Chun, J. H., Dauparas, J., Bennett, N., Wicky, B. I. M., Muenks, A., DiMaio, F., Correia, B., Ovchinnikov, S. & Baker, D. (2022). *Science*, **377**, 387–394. 10.1126/science.abn2100 PMC9621694 35862514

[bb56] Wei, R., von Haugwitz, G., Pfaff, L., Mican, J., Badenhorst, C. P. S., Liu, W., Weber, G., Austin, H. P., Bednar, D., Damborsky, J. & Bornscheuer, U. T. (2022). *ACS Catal.* **12**, 3382–3396. 10.1021/acscatal.1c05856 PMC8939324 35368328

[bb57] Wollenhaupt, J., Barthel, T., Lima, G. M. A., Metz, A., Wallacher, D., Jagudin, E., Huschmann, F. U., Hauss, T., Feiler, C. G., Gerlach, M., Hellmig, M., Förster, R., Steffien, M., Heine, A., Klebe, G., Mueller, U. & Weiss, M. S. (2021). *J. Vis. Exp.* **169**, e62208. 10.3791/62208 33749678

[bb58] Wollenhaupt, J., Metz, A., Barthel, T., Lima, G. M. A., Heine, A., Mueller, U., Klebe, G. & Weiss, M. S. (2020). *Structure*, **28**, 694–706.e5. 10.1016/j.str.2020.04.019 32413289

[bb59] Yasuhira, K., Shibata, N., Mongami, G., Uedo, Y., Atsumi, Y., Kawashima, Y., Hibino, A., Tanaka, Y., Lee, Y.-H., Kato, D., Takeo, M., Higuchi, Y. & Negoro, S. (2010). *J. Biol. Chem.* **285**, 1239–1248. 10.1074/jbc.M109.041285 PMC2801252 19889645

[bb61] Yoshida, S., Hiraga, K., Takehana, T., Taniguchi, I., Yamaji, H., Maeda, Y., Toyohara, K., Miyamoto, K., Kimura, Y. & Oda, K. (2016). *Science*, **351**, 1196–1199. 10.1126/science.aad6359 26965627

[bb62] Zhang, L., Lin, D., Sun, X., Curth, U., Drosten, C., Sauerhering, L., Becker, S., Rox, K. & Hilgenfeld, R. (2020). *Science*, **368**, 409–412. 10.1126/science.abb3405 PMC7164518 32198291

